# Positive Effect of Lecithin-Based Delivery Form of Curcuma and Boswellia Extracts on Irritable Bowel Syndrome After COVID-19 Infection

**DOI:** 10.3390/nu17040723

**Published:** 2025-02-18

**Authors:** Attilio Giacosa, Gaetan Claude Barrile, Clara Gasparri, Simone Perna, Mariangela Rondanelli

**Affiliations:** 1 Italian Diagnostic Center (CDI), 20147 Milan, Italy; attilio.giacosa@gmail.com; 2 Endocrinology and Nutrition Unit, Azienda di Servizi Alla Persona “Istituto Santa Margherita”, University of Pavia, 27100 Pavia, Italy; clara.gasparri01@universitadipavia.it; 3 Department of Food, Environmental and Nutritional Sciences, Division of Human Nutrition, University of Milan, 20133 Milan, Italy; simoneperna@hotmail.it; 4 Department of Public Health, Experimental and Forensic Medicine, University of Pavia, 27100 Pavia, Italy; mariangela.rondanelli@unipv.it

**Keywords:** abdominal pain, bloating, *Boswellia serrata*, COVID-19 infection, *Curcuma longa*, dysbiosis, IBS (irritable bowel syndrome), long COVID

## Abstract

**Background**: Post-COVID-19 irritable bowel syndrome (PCIBS) is a frequent finding and is frequently associated with enteral dysbiosis. This pilot study compared the effects of extracts from curcuma and boswellia on PCIBS and irritable bowel syndrome (IBS) in individuals who had never had a COVID-19 infection (controls). **Methods**: A total of 16 subjects with PCIBS and 28 controls with evidence of IBS gastrointestinal symptoms and with enteral dysbiosis were recruited and supplemented for 30 days with sunflower-lecithin-based formulations of extracts of *Curcuma longa* (500 mg) and *Boswellia serrata* (150 mg) b.i.d. and with low-FODMAP diet. Abdominal bloating, abdominal pain, enteral dysbiosis (as increased urinary indican), and the global assessment of efficacy (GAE) were evaluated at the end of the study. **Results**: In both cohorts, intra-cohort changes revealed a statistically significant (*p* < 0.05) reduction in bloating and abdominal pain. The GAE showed similar and relevant satisfactory rates in both groups. On the contrary, urinary indican values showed a significant decrease only in the IBS group. **Conclusions**: Supplementation with Curcuma and Boswellia has favorable effects on abdominal bloating and abdominal pain of subjects with PCIBS and with IBS, while enteral dysbiosis is significantly decreased only in patients with IBS. Additional studies are needed to confirm these preliminary findings and to clarify the reasons for the persistence of dysbiosis in PCIBS.

## 1. Introduction

COVID-19 infection is a disease mainly associated with severe acute respiratory failure. This infectious disease began in China in 2019 and has spread throughout the world in subsequent years. The World Health Organization stated that at the end of March 2024, over 774 million people had had this disease and more than seven million had died [1]

It has been demonstrated that SARS-CoV-2 may be found in the gastrointestinal tract (GIT) and that the angiotensin-converting enzyme 2 (ACE2) receptor is highly expressed throughout the GIT. Consequentially, SARS-CoV-2 may enter GIT cells via ACE2 receptors and damage the GIT organs [2]. SARS-CoV-2 was observed in the colonic tissues and feces of patients with COVID-19 [3]. In a meta-analysis of 60 studies comprising 4243 patients, the pooled prevalence of all gastrointestinal symptoms was 17.6% [4].

Weng and colleagues evaluated the long-term GIT pathological consequences of 117 patients hospitalized for COVID-19 infection [3]. Fifty-two (44%) patients reported gastrointestinal symptoms 90 days after discharge. The most frequent gastrointestinal symptoms were loss of appetite, nausea (18%), acid reflux (18%), diarrhea (15%), abdominal bloating (14%), belching (10%), vomiting (9%), and abdominal pain (7%). Various researchers, after having observed the appearance of new gastrointestinal symptoms at 6 months following COVID-19 infection, hypothesized the presence of a post-COVID-19 irritable bowel syndrome (PCIBS) [5,6].

A retrospective review of the clinical data of 147 COVID-19 patients found new GI symptoms in 16% of them, at a median follow-up time of 106 days [5]. The same study showed that 40% of the COVID-19 survivors reported new GI symptoms at 6 months [5]. These results have been confirmed by an international epidemiologic study on 614 COVID-19 patients, showing that at the 12-month follow-up, patients with COVID-19 had significantly higher rates of IBS than patients in the control group [7]. Moreover, gut dysbiosis has been described for at least 6 months in patients with PCIBS [8].

Recent reviews have evaluated the effects of various dietary supplements on gastrointestinal disorders, intestinal dysbiosis, and related symptoms [9,10,11]. Our research group demonstrated the presence of enteral dysbiosis in IBS patients with abdominal bloating as a prevalent finding and reported that supplementation with Curcuma and Boswellia extracts, as sunflower-lecithin-based formulations at different dosages, was efficacious in significantly reducing both the dysbiosis and abdominal bloating when combined with a low-FODMAP diet (LFD). This effect was significantly better when compared with a LFD alone [12].

These beneficial effects might result from the anti-inflammatory and gut antimicrobial modulation properties [12,13,14,15,16,17,18] exerted by Boswellia and Curcuma extracts [12,19,20]

Based on these findings, the purpose of this pilot study was to compare this cohort with subjects with IBS who had not previously shown signs of COVID-19 infection and to confirm the effects of the combination of Curcuma and Boswellia, both of which were formulated separately in phospholipids (as Meriva^TM^ and Casperome^TM^), along with an LFD regimen, in subjects with post COVID-19 infection with IBS-like symptoms, with small-bowel dysbiosis and abdominal bloating being the most relevant symptoms [21,22].

## 2. Materials and Methods

### 2.1. Study Design

This was a 30-day-supplementation case study observation in which two different groups of participants were compared, i.e., subjects with long COVID and IBS-like symptoms (the PCIBS group) and subjects with a diagnosis of IBS without previous COVID-19 infection (the control group). This two-cohort open-label study was conducted at the University of Pavia’s Department of Public Health in Italy. The study’s goal was to assess and contrast the safety and effectiveness of Curcuma and Boswellia extracts, prepared separately in phospholipids, in individuals with or without long-term COVID-19 infection with symptoms of IBS, abdominal bloating, and enteral dysbiosis. After receiving approval from the local independent ethics committee, the study was carried out in compliance with the ICH Guidelines for Good Clinical Practice and the Declaration of Helsinki (Ethic code number: 0912/09052020). Written informed consent was obtained from each participant. The study was conducted from 1 September 2021 to 10 June 2022.

### 2.2. Population

Male and female subjects, aged 18–75 years, with a diagnosis of PCIBS, 60–120 days after the end of their COVID-19 infection, were recruited, together with subjects of the same age with IBS without previous COVID-19 infection (the control group). The inclusion criteria for both PCIBS and IBS (Control) groups were as follows: (1) age: 18–75 years, male/female; (2) evidence of functional abdominal bloating/distention (FAB/D)-type IBS, according to Lacy et al. [11]; (3) presence of enteral dysbiosis, defined by the increase in urinary indican values with a normal skatole urinary concentration [12,23,24]; (4) absence of gastroenterological treatments in the last 15 days before starting the therapeutic intervention, except antispasmodics and anxiolytics.

The exclusion criteria for both PCIBS and controls were as follows: (1) normal urinary indican values or increased urinary skatole values; (2) subjects already on a LFD or other dietary restrictions, such as a gluten-free diet or lactose-free diet, within the past 6 months; (3) insulin-dependent diabetes; (4) known history of microscopic colitis, inflammatory bowel illness, diverticular disease, or celiac disease; (5) previous cholecystectomy or small-bowel or colonic surgery; (6) severe vomiting or bloody diarrhea; (7) hepatic disease (defined as altered values of liver function tests) or severe renal disease (defined as serum creatinine > 1.5 mg/dL).

### 2.3. Supplementation and Concomitant Medications

All subjects (in the PCIBS and control groups) included in the study received film-coated tablets twice daily for 30 days, each containing a combination of 500 mg of *Curcuma longa* L. as a sunflower-lecithin-based formulation (Meriva™) and 150 mg of *Boswellia serrata* extract as a sunflower-lecithin-based formulation (Casperome™), which were kindly provided by Indena S.p.A., Milan, Italy. Meriva is a food-grade lecithin formulation of curcumin in 500 mg film-coated tablets, containing a standardized amount of 100 mg of highly bioavailable curcuminoids [21]. Casperome is a delivery form of a highly standardized *Boswellia serrata* extract and soy lecithin in a 1:1 ratio. [22]. The choice of dose was based on previous clinical experiences with Curcuma and Boswellia extracts and the daily suggested dosage of each single botanical ingredient [12,25].

The ratio of the amount of supplement consumed (as shown by the pills that were returned) to the anticipated consumption for each participant throughout the actual supplementation period was used to estimate the supplementation compliance. All participants (in the PCIBS and control groups) received dietary guidance from the same dietician regarding a lLFD [26]. Concurrent administration of any other medications for the treatment of digestive disorders that might influence the results or interfere with the study supplementation was prohibited. These medications included anxiolytics, short-acting analgesics (like paracetamol), and short-acting spasmolytics (like butylscopolaminium bromide, dihydrate phloroglucinol, and derivatives).

### 2.4. Clinical Evaluation

On the questionnaire, participants were asked whether they felt “bloated/uncomfortably full”. There were four options to choose from: none (symptom did not occur) (score: 0); mild (symptom occurred but did not interfere with usual activities) (score: 1); moderate (occurrence of symptom somewhat interfered with usual activities) (score: 2); or severe (occurrence of symptom resulted in an inability to perform usual activities) (score: 3) [27]. An established visual analog scale for measuring pain was used to determine the level of the abdominal pain (0 being “no pain” and 10 being “most severe pain”) [28,29].

Urinary indican and skatole levels were measured for each patient seven days before study participation and at the conclusion of the study to determine intestinal dysbiosis (29). Skatole and indican levels in the urine were regarded as normal when they were less than 10 µg/L and 10 mg/L, respectively [30]. Every participant completed a global assessment of efficacy (GAE) using a 4-point scale at the conclusion of the study with 1 denoting “ineffective”; 2 being “moderately effective/slight improvement in complaints”; 3 denoting “effective/marked improvement in symptoms”; and 4 being “very effective/as good as no symptoms”, according to Kruis and colleagues [31,32]. Serum and urine samples were taken for safety monitoring at every appointment, and vital signs were examined. Analyses were conducted in the laboratory and compared to the usual ranges. During the 30-day supplementation period, participants were told to report any discomfort as soon as possible, as this was considered to be an adverse event. At the last appointment, adverse events were also examined.

### 2.5. Study Endpoints

The reduction in the severity of stomach bloating was the primary outcome. The following were secondary endpoints: (1) the change in urine indican levels; (2) the change in the intensity of stomach discomfort; and (3) the GAE, as determined by the participants at the conclusion of the research. Laboratory findings, vital signs, and adverse occurrences were all considered safety endpoints.

### 2.6. Statistical Analysis

Baseline data have been presented as the mean values ± standard deviation of the mean (SD) unless otherwise indicated. The normal distribution of the variables was checked using the Shapiro–Wilk test and using Q–Q graphs. Baseline differences in demographic and clinical characteristics between the two groups (PCIBS and control) were examined using independent *t*-tests.

The homogeneity of the variances was estimated by using Levene’s test. A one-factor covariance ANCOVA test was used for continuous variables to determine differences between the two cohorts.

The same model was used to compare changes in the variables regarding the effects between the two cohorts: the effect was quantified by no infection (control) minus infection (PCIBS), adjusting for age and gender. Correlations between changes pre- and post-study in the PCIBS and control groups have been estimated by the Pearson’s correlation coefficient when the assumptions of normality were met and by the Spearman’s correlation coefficient when the assumptions of normality were not met. A *p* < 0.05 value was considered significant. The Statistical Package for the Social Sciences version 28 software was used to perform the statistical analysis (SPSS Inc., Chicago, IL, USA).

## 3. Results

**Figure 1:**
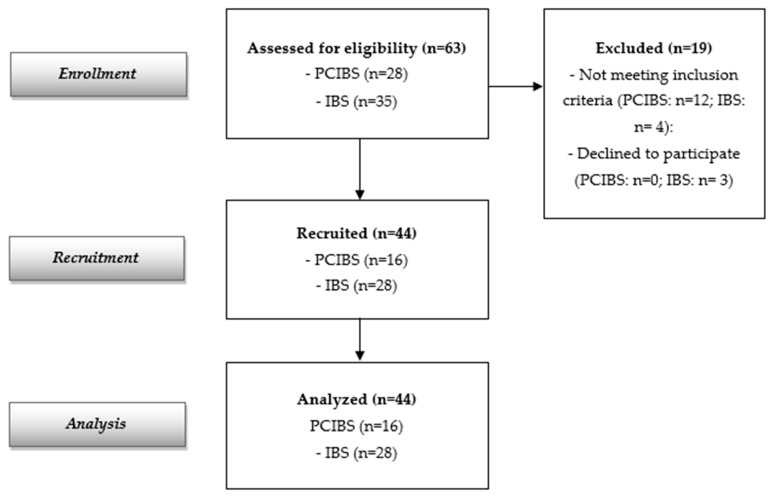
Flow diagram of the study.

The flow diagram reported in Figure 1 indicates 63 screened participants (28 with PCIBS and 35 subjects with IBS without previous COVID-19 infection). Forty-four subjects were recruited and analyzed (16 with PCIBS and 28 subjects with IBS without previous COVID-19 infection, considered as the control group). The recruited population at baseline is described in Table 1. The data show a total of 44 recruited adults, 28 females and 16 males (mean age: 44.59 ± 16.20 years); 16 were in the PCIBS group and 28 were in the control group. All of them completed the study. Baseline demographic and clinical characteristics were similar in both groups. Only the abdominal bloating score differed at baseline between the two cohorts, although this was the major symptom in both groups.

No adverse effects related to the supplementation were reported during the study.

Participants in the control group had less diarrhea (39.3%) compared with the PCIBS group at baseline (62.5%). Regarding the incidence of constipation, the two cohorts showed similar data (control group: 35.7% versus PCIBS group: 25%), and there has been no evidence of a statistically significant correlation between the two groups or of constipation or diarrhea.

The mean difference changes in the primary and secondary outcomes after supplementation are reported in Table 2.

Intra-cohort changes showed a statistically significant (*p* < 0.05) decrease in bloating in both cohorts. Also, the abdominal pain decreased significantly in both groups. On the contrary, urinary indican values showed a significant decrease only in the control group (subjects with IBS without previous COVID-19 infection).

**Figure 2:**
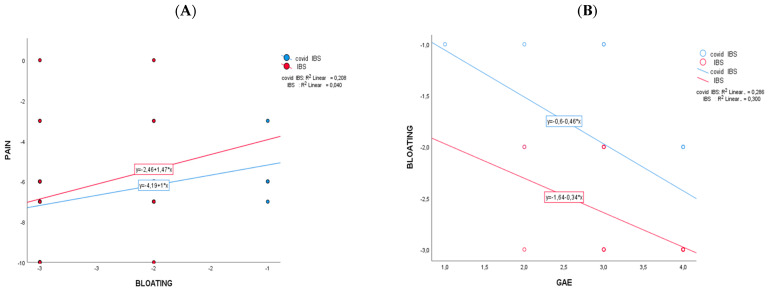
Pearson correlations between Δ-changes (end vs. baseline) on the two cohorts that changed significantly. In (**A**) is pain versus bloating and in (**B**) is bloating versus GAE.

The comparison between the two cohorts (net effect in control minus PCIBS group) showed that the changes differ significantly for bloating (−0.899; CI95%: −1.270; −0.528; *p* < 0.001) and for urinary indican values (−44.090; CI95%: −74.551; −13.629; *p* = 0.006). No significant changes between the cohorts have been recorded for abdominal pain (Table 2) and GAE (Table 3). After supplementation (Table 3), 81.3% of participants in the PCIBS group has a GAE equal to or higher than grade 3, as a sum of grade 3 (“effective/marked improvement in symptoms”) and grade 4 (“very effective/as good as no symptoms”); among the control group, the value was 92.8%. Figure 2 reports the results of the Pearson correlation analysis of mean difference changes (t1−t0) with the intervention regarding the statistically significant markers of Table 2. The correlation scatterplot showed that in the PCIBS cohort, the decrease over time in pain was significantly correlated with the decrease in abdominal bloating (r = 0.456; *p* > 0.01) (Figure 2A). A negative statistically significant association was recorded in both cohorts between a decrease in bloating over time and an increase in the GAE score at the end of the study (r2 = −0.547, control group; and r2 = −0.532, PCIBS group; *p* > 0.01) (Figure 2B).

## 4. Discussion

This study demonstrates that supplementation with Curcuma and Boswellia extracts combined with a low-FODMAP diet is correlated with a significant decrease in bloating in subjects with long COVID and FAB/D-type IBS-like symptoms (PCIBS); this is similar to what was found in subjects with FAB/D-type IBS without previous COVID-19 viral infection [11]. These findings prove that supplementation of a rational premix combination of Curcuma phospholipids and Boswellia phospholipids is efficacious in achieving the primary endpoint of the study, that is, the decrease in abdominal bloating in subjects with FAB/D-type IBS, with or without previous COVID-19 infection.

In our trial, positive effects were also been found when abdominal pain was evaluated. A significant relief of abdominal pain was observed in the PCIBS and control groups (IBS without previous COVID-19 infection).

These results suggest that the combination of Curcuma and Boswellia extracts may be a promising supplement to treat abdominal bloating and abdominal pain in subjects with PCIBS and in subjects with IBS without previous COVID-19 infection.

A previous study of subjects with IBS without previous COVID-19 infection showed that these results are specifically due to the Curcuma and Boswellia phospholipid-based supplement [12]. In that clinical observation, the control group with IBS followed only an LFD regimen with significantly lower efficacy compared to the IBS group supplemented with Curcuma and Boswellia extracts in association with an LFD [12].

The favorable outcome of this rational botanical supplementation is confirmed by the participants’ global assessment of efficacy that showed a similarly positive outcome in both of the groups. At the end of the study, 81.3% of participants in the PCIBS group were classified as grade 3 (“effective/marked improvement in symptoms”) or grade 4 (“very effective/as good as no symptoms”) in terms of the GAE, and this result was similarly obtained by 92.8% of participants in the control group (subjects with IBS without previous COVID-19 infection).

A correlation analysis confirmed the relevant importance of treating abdominal bloating by showing that in both the cohorts, the decrease over time in pain was significantly correlated with the decrease in bloating. In addition, a statistically significant association was recorded in both cohorts between a decrease in bloating over time and an increase in the GAE score at the end of study. Additional studies are needed to confirm this preliminary observation and to evaluate the long-term effect of this treatments in subjects with IBS.

Post-infectious IBS is a well-known clinical finding. Stewart was the first to describe this phenomenon in 1950 [33]. In these cases, gastrointestinal symptoms may persist following clearance of an infecting intestinal pathogen. The COVID-19 pandemic has overwhelmed healthcare services since 2019 and acute gastrointestinal symptoms have been frequently found in addition to the usual respiratory problems [34]. Moreover, prospective studies showed the appearance and persistence of symptoms compatible with IBS diagnostic criteria various months after serological negativization of the infection [3,6,35].

A recent review hypothesized various mechanisms for the pathogenesis of post-acute-COVID-19 irritable bowel syndrome (PCIBS), including persistent inflammation, autoimmunity, viral antigen persistence, altered cytokine production, prior mental health conditions, maladaptive neuro-immune interactions, and alteration to the fecal microbiome [36]. Multiple evidences suggest that oxidative stress plays a critical role in the pathophysiology of COVID-19 infection and the long-COVID condition, and that after the acute phase of COVID-19 infection, the disease is dominated by immune–pathological pro-inflammatory elements [37,38]. The anti-inflammatory effect of both Curcuma and Boswellia extracts could at least, in part, explain the benefit of this supplementation in subjects with long-COVID-19 and IBS-like symptoms [13,39,40,41,42].

Various factors that promote epithelial barrier damage, intestinal inflammation, gut dysfunction, and intestinal dysbiosis (such as antibiotics and other pharmacological treatments of the acute phase of COVID-19 infection, gut–lung axis impairment, disease-related psychological stress, as well as the virus itself) could be involved in the pathogenic process of PCIBS [43].

As has previously been reported, Qin Liu and colleagues showed that gut dysbiosis persists for at least 6 months in patients with post-acute-COVID-19 syndrome [8]. The gut microbiomes of these patients are characterized by higher levels of *Ruminococcus gnavus* and *Bacteroides vulgatus*, and lower levels of *Faecalibacterium prausnitzii*. Butyrate-producing bacteria, including *Bifidobacterium pseudocatenulatum* and *Faecalibacterium prausnitzii*, showed the largest inverse correlations with post-acute-COVID-19 syndrome at 6 months [8]. Based on these observations, the possible presence of small-bowel dysbiosis was evaluated in the enrollment phase of this study and was confirmed in 24 out of 28 post-COVID-19 patients with IBS-like symptoms (85.7%). Supplementation with a combination of Curcuma phospholipids and Boswellia phospholipids produced a significant difference when the effect on urinary indican of subjects with PCIBS and subjects with IBS without previous COVID-19 infection were compared. As a matter of fact, a significant reduction in urinary indican, as the marker of small-bowel dysbiosis, was observed only in subjects with IBS without COVID-19 infection. Meanwhile subjects with PCIBS did not show significant urinary indican changes after supplementation. In addition, when the two cohorts were compared, the indican difference achieved a statistically significant value. These data are correlated with the previous results reported by Qi Su and colleagues, who showed that in subjects with post-acute-COVID-19 gastrointestinal symptoms, gut dysbiosis may linger beyond one year after SARS-CoV-2 clearance [44]. To date, there has been no clarity on the reason for this behavior. It was reported that Curcuma extracts favor beneficial bacterial strains in the gut microbiota [45]. Interestingly, two distinct phenomena that may be related to curcumin activity are produced by the interaction between curcumin and gut flora. Curcumin’s beneficial modulation of intestinal microflora and the gut microbiota’s biotransformation of curcumin serve as examples of these two phenomena [45]. In addition, *Boswellia serrata* resin has been added as a supplement to rabbit diets at different dosages to obtain changes in the fecal microbiota. Relevant changes were found in the cecal microbiota of rabbits treated with *Boswellia serrata*, with a significant reduction in bacterial counts and, in particular, a decrease in *Salmonella enteritidis* and *Escherichia coli*, compared to the untreated control group. These results could be due to the high polyphenol content of *Boswellia serrata* extracts and to the presence of boswellic acids, which have a powerful antimicrobial effect [46]. Therefore, both *Curcuma longa* and *Boswellia serrata* extracts may act favorably on gut microbiota and intestinal dysbiosis, as clearly demonstrated in the subjects with IBS without previous COVID-19 infection who were evaluated in this study. On the contrary, this did not occur in subjects with PCIBS, thus confirming the need of additional research on the pathogenesis and biological characteristics of PCIBS. Even though the intra-cohort analysis showed a significant reduction in bloating after supplementation in both cohorts, the effect of supplementation was more prominent in the control group as compared to the PCIBS cohort, and this could possibly be influenced by the persistence of dysbiosis in the latter group, as shown also by Meringer and Mehandru [36].

Phytosome™ technology is a food-grade delivery system in the form of a lecithin-based solid dispersion of botanical ingredients with the aim of improving their solubility in gastro-intestinal fluids and promoting their effectiveness. Bresciani et al. recently showed that the formulation of phytosomes significantly affected the biotransformation of curcuminoids because the fecal human microbiota fermented lecithin–curcuminoids, which resulted in the more effective production of curcuminoid catabolites [47]. In our study, the Phytosome technology has been used in both Curcuma and Boswellia extracts and this could have promoted the favorable clinical outcome, due to a potential benefit of the highly bioavailable extracts.

This study aimed to explore whether a shared intervention (Curcuma/Boswellia + a low-FODMAP diet) could alleviate overlapping symptoms (bloating, pain) in post-COVID-19 IBS (PCIBS) and traditional IBS, despite differing origins. While both groups showed symptomatic improvement, the lack of urinary indican reduction in people with PCIBS suggests divergent dysbiosis mechanisms compared to those with traditional IBS, implying that while anti-inflammatory effects may address shared symptoms, PCIBS may require etiology-specific strategies for dysbiosis. The comparison highlights the intervention’s potential for symptom relief across etiologies but underscores the need for further research to unravel the specific pathophysiology of PCIBS.

## 5. Limitations

Various limitations of our preliminary study need to be considered. One of the study’s weaknesses is the absence of a post-COVID-19 randomized control group with IBS-like symptoms who received a placebo and another group who received only an LFD. An additional limitation is that a group of patients with PCIBS with normal urinary indican values has not been considered. Moreover, none of the study groups’ adherence to an LFD has been examined. This is important mostly because the LFD is considered to be the first-line standard of care in the management of GI symptoms in IBS patients. However, further studies should possibly address those aspects.

## 6. Conclusions

In conclusion, this study shows positive effects of supplementation with *Curcuma longa* and *Boswellia serrata* extracts (as Curcuma phospholipids and Boswellia phospholipids) on the abdominal bloating and abdominal pain of subjects with post-acute-COVID-19 IBS-like symptoms. These results are similar to those observed in subjects with IBS without previous COVID-19 infection. In addition, the results of this investigation may indicate that small-bowel dysbiosis is a frequent finding in people with post-acute-COVID-19 infection and that it remains following supplementation with extracts of Curcuma longa and Boswellia serrata, but it drastically diminishes in participants with IBS without previous COVID-19 infection. Further research is required to validate these results, elucidate the pathophysiology of post-acute PCIBS, and assess the causes of the persistence of small-bowel dysbiosis following supplementation with extracts of Boswellia serrata and Curcuma longa.

## Figures and Tables

**Table 1:** Descriptive statistics of the sample at baseline.

Variable	Control (Mean ± SD)	PCIBS (Mean ± SD)	*p*-Value Between Cohorts at Baseline
Number (M/F)	28 (7 M, 21 F)	16 (9 M, 7 F)	
Age (years)	42.21 ± 14.71	48.75 ± 18.27	0.202
BMI	24.31 ± 2.14	25.13 ± 2.37	0.206
Time with disease (months)	3.28 ± 0.60	4.07 ± 0.85	0.128
Abdominal Bloating	2.96 ± 0.18	2.63 ± 0.61	0.010
Urinary Indican	90.57 ± 45.40	75.19 ± 32.45	0.241
Abdominal Pain (scale 0–10)	7.57 ± 2.00	7.50 ± 2.78	0.929
Diarrhea Yes	39.3%	62.5.%	0.138
Constipation Yes	35.7%	25.0%	0.463

**Table 2:** Mean difference changes in primary and secondary outcomes at the end of study.

Variable	Control Intra-Group Δ Change (CI 95%)	PCIBS Intra-Group Δ Change (CI 95%)	Δ Change Differences Between Cohorts (CI 95%)	*p*-Value Between Cohorts
Abdominal Bloating	−2.850 (−3.063; −2.637)	−1.950 (−2.238; −1.662)	−0.899 (−1.270; −0.528)	**0.001**
Urinary Indican	−36.212 (−53.713; −18.711)	7.878 (−15.775; 31.528)	−44.090 (−74.551; −13.629)	**0.006**
Abdominal pain	−6.552 (−7.511; −5.593)	−6.284 (−7.560; −5.008)	−0.268 (−1.879; 1.373)	0.793

In bold: value with *p* < 0.05.

**Table 3:** Frequencies of GAE (global assessment of efficacy) at the end of study.

GAE Score	PCIBS Group	Control (IBS) Group	*p*-Value
1 (n)	1	0	>0.05
%	6.3%	0.0%	
2(n)	2	2	
%	12.5%	7.1%	
3 (n)	8	9	
%	50.0%	32.1%	
4 (n)	5	17	
%	31.3%	60.7%	
Total (n)	16	28	
%	100.0%	100.0%	

## Data Availability

All data generated or analyzed during this study are included in this published article. The data supporting this study are available from the corresponding author upon reasonable request.
